# Supplementary material for: Genome-wide identification of the CYP82 gene family in cucumber and functional characterization of CsCYP82D102 in regulating resistance to powdery mildew
Source: PeerJ. 2024 Mar 28;12:e17162. doi: 10.7717/peerj.17162 (PMC10981884; doi:10.7717/peerj.17162)
Supplement: Supplemental Information 1 [file peerj-12-17162-s001.docx]

**Table S1.** The primer sequence.

| **Primer comments** | **Primer sequence** |
| --- | --- |
| *CYP82D102* cloning | F: ATGGAACCGCTGTTTGCTTTA  R: ACTTTATAATCTTGTGCTGATAGGCG |
| qRT-PCR of *CYP82D102* | F: CATGCCATTGGCGATGATTGTCATACGCAA  R: GGAAGATCTGATAATTCATAAACTTGAGGTGG |
| Csa3G852580 | F: ACCGAGGATTGCACAATTGG  R: TCCGGTTTGAACTTTGAAGGC |
| Csa3G852560 | F: TGCCCGTAAACGCAAAGAAC  R: AGGCTCCTAAGGTTTGATGAGG |
| Csa3G852610 | F: ATCTCGCCCGAAATTTGCTG  R: AGCCTGTTCATACGGTGGTTAG |
| Csa3G853140 | F: AATAGCCACACTCGAACTCCTG  R: AAGTTCCTCCATCCATGTCTCG |
| Csa3G852600 | F: CCCGAGCGATTTTTGACAAC  R: ATGCAAGAACCCAGCCAAAG |
| Csa3G852590 | F: AATCCATCACCATGCTCGTC  R: TGTACTGTTGCCACTTCTGC |
| Csa3G852640 | F: TGCTCGTCGAAACAAACTCC  R: TATGTGCGCTGGTTCTGTTG |
| Csa3G852630 | F: CGCCATGTTCGCAAGTTAAC  R: TTGGACCTCGGATACCCTTATG |
| Csa3G853160 | F: AAACTGCAACCACCTGAAGC  R: ATTGGTCCATGAGCATCTGC |
| NPR1 | F: CGACGGTATTTCCCTCATTG  R: CTTCTTCCGCTGCTCTTCTG |
| PR1 | F: TGGAGAAATACGCAAAGGATGG  R: CAGGCGGATCATAGTTACAAGTCA |
| PR2 | F: TGGTCACTGCAACCCTGACA  R: AGTGGCCTGGAATCCGACT |
| PR4 | F: ATACGGTTGGACTGCCTTCTGT  R: CGAAGCTCCCGTTTCAGTGTTAG |
| PR5 | F: CATCCGGACACGGCAGCT  R: TCCATGTACTGCTTCAGAGCG |
| LOX2 | F: AAGGTTTGCCTGTCCCAAGA  R: TGAGTACTGGATTAACTCCAGCCAA |
| JAR1 | F: CCTTTGCTGTTCTACCGA  R: TACACTTCACCGATCTTGAC |
| CAT2 | F: TCAAACCATGGATCCTTACAAGT  R: TGTTCCATACAGGAGCACCA |
| APX | F: GCAGATCTGTACCAGCTTGCTG  R: CAGCAATTCCACAAAGTAGGAGTTATC |
| *CsACTIN* | F: ACGCTGTTGGTGGTGGTAC  R: GAAAGGGGTAAACAGTGAATC |
