# Supplementary material for: Genome-wide identification of the CYP82 gene family in cucumber and functional characterization of CsCYP82D102 in regulating resistance to powdery mildew
Source: PeerJ. 2024 Mar 28;12:e17162. doi: 10.7717/peerj.17162 (PMC10981884; doi:10.7717/peerj.17162)
Supplement: Supplemental Information 2 [file peerj-12-17162-s002.docx]

**Table S2.** Basic information of members of CYP82 gene family in cucumber.

| **Gene ID** | **Naming** | **MW (kda)** | **pI** | **Protein length(aa)** | **Subcellular Localization** |
| --- | --- | --- | --- | --- | --- |
| Csa3G852560 | CYP82D98 | 65839.3 | 7.94 | 587 | Endoplasmic reticulum |
| Csa3G852580 | CYP82D99 | 60444.7 | 7.85 | 535 | Endoplasmic reticulum |
| Csa3G852590 | CYP82AG1 | 60288.3 | 8.44 | 532 | Endoplasmic reticulum |
| Csa3G852600 | CYP82AG2 | 60387.3 | 9.17 | 532 | Endoplasmic reticulum |
| Csa3G852610 | CYP82D101 | 40058 | 7.99 | 347 | Endoplasmic reticulum |
| Csa3G852620 | CYP82D104 | 60593.7 | 8.28 | 526 | Endoplasmic reticulum |
| Csa3G852630 | CYP82D102 | 61651.1 | 8.54 | 538 | Endoplasmic reticulum |
| Csa3G852640 | CYP82D103 | 59729.8 | 8.38 | 521 | Endoplasmic reticulum |
| Csa3G853140 | CYP82D100 | 36767.9 | 9.52 | 315 | Endoplasmic reticulum |
| Csa3G853150 | CYP82D105 | 60509.4 | 7.43 | 529 | Endoplasmic reticulum t |
| Csa3G853160 | CYP82D106 | 59638.4 | 6.85 | 529 | Endoplasmic reticulum |
| Csa3G853170 | CYP82D107 | 61107.5 | 8.28 | 535 | Endoplasmic reticulum |
