# Supplementary material for: Genome-wide identification of the CYP82 gene family in cucumber and functional characterization of CsCYP82D102 in regulating resistance to powdery mildew
Source: PeerJ. 2024 Mar 28;12:e17162. doi: 10.7717/peerj.17162 (PMC10981884; doi:10.7717/peerj.17162)
Supplement: Supplemental Information 3 [file peerj-12-17162-s003.docx]

**Table S3.** Motif information of members of gene family

| **Motif** | **E-value** | **sites** | **Width** | **Sequence** |
| --- | --- | --- | --- | --- |
| 1 | 1.20E-263 | 12 | 41 | ADAYGPIFTLRLGMHRALVVSNWEIAKECFTTNDRIFASRP |
| 2 | 2.40E-250 | 10 | 50 | PRESTEDCTIAGYHIPAGTRLIVNIQKJQRDPRVWEDPCEFKPERFLTSH |
| 3 | 1.90E-227 | 12 | 50 | RDFFELFGVFVPSDSFPFLSWLDLGGYEKAMKKTAKILDEVLDKWLEEHR |
| 4 | 9.50E-216 | 10 | 50 | QNPZLIPFGSGRRMCPGLSFALQMMHLALANLLHGFEIKRPSKELIDMEE |
| 5 | 1.20E-229 | 10 | 50 | NNEEALKKAQLELDEQVGRQRQVKESDIKNLVYLQAIVKETLRLYPAGPL |
| 6 | 1.90E-180 | 11 | 41 | KLVASKLLGYBYAMFGLSPYGPYWRHIRKIAMLELLSNHRL |
| 7 | 1.60E-122 | 12 | 29 | KVLVEMKKWFGDITLNTIFKMVIGKRFST |
| 8 | 2.70E-100 | 12 | 21 | PPEPGGAWPVIGHLHLLNASE |
| 9 | 2.90E-94 | 11 | 35 | LSLVDDDEZLSGYDADTVIKATCLALILGGTDTTT |
| 10 | 8.90E-53 | 12 | 21 | QLQHIRVSEVQTSIKELYELW |
