# Supplementary material for: Genome-wide identification of the CYP82 gene family in cucumber and functional characterization of CsCYP82D102 in regulating resistance to powdery mildew
Source: PeerJ. 2024 Mar 28;12:e17162. doi: 10.7717/peerj.17162 (PMC10981884; doi:10.7717/peerj.17162)
Supplement: Supplemental Information 4 [file peerj-12-17162-s004.docx]

**Table S4.** Comparison of A-type P450 families among cucumber, Arabidopsis, Tomato, soybean, Maize, rice, poplar, grape, and moss

| A-Type Family | Cucumber | Arabiodopsis | Tomato | Soybean | Maize | Rice | Popular | Grape | Moss |
| --- | --- | --- | --- | --- | --- | --- | --- | --- | --- |
| **CYP71 Clan** |  |  |  |  |  |  |  |  |  |
| CYP71 | 3 | 52 | 42 | 55 | 56 | 84 | 25 | 24 | 0 |
| CYP73 | 2 | 1 | 1 | 3 | 3 | 3 | 3 | 3 | 4 |
| CYP75 | 4 | 1 | 2 | 7 | 5 | 3 | 3 | 11 | 0 |
| CYP76 | 7 | 8 | 18 | 14 | 6 | 29 | 13 | 24 | 0 |
| CYP77 | 3 | 5 | 3 | 4 | 2 | 2 | 3 | 2 | 0 |
| CYP78 | 5 | 6 | 5 | 11 | 8 | 8 | 10 | 7 | 3 |
| CYP79 | 4 | 7 | 5 | 5 | 4 | 4 | 4 | 9 | 0 |
| CYP80 | 0 | 0 | 5 | 0 | 0 | 0 | 6 | 6 | 0 |
| CYP81 | 18 | 18 | 11 | 12 | 12 | 12 | 28 | 21 | 0 |
| CYP82 | 12 | 5 | 9 | 24 | 0 | 0 | 10 | 34 | 0 |
| CYP83 | 0 | 1 | 0 | 12 | 0 | 0 | 5 | 0 | 0 |
| CYP84 | 2 | 2 | 1 | 3 | 2 | 3 | 3 | 3 | 0 |
| CYP89 | 10 | 7 | 2 | 8 | 16 | 14 | 10 | 14 | 0 |
| CYP92 | 0 | 0 | 7 | 2 | 11 | 9 | 8 | 6 | 0 |
| CYP93 | 1 | 1 | 1 | 13 | 7 | 3 | 4 | 4 | 0 |
| CYP98 | 1 | 3 | 3 | 2 | 2 | 2 | 5 | 1 | 1 |
| CYP99 | 0 | 0 | 0 | 0 | 4 | 2 | 0 | 0 | 0 |
| CYP701 | 1 | 1 | 1 | 2 | 2 | 5 | 1 | 1 | 1 |
| CYP703 | 1 | 1 | 1 | 1 | 1 | 1 | 1 | 1 | 3 |
| CYP705 | 0 | 26 | 0 | 0 | 0 | 0 | 0 | 0 | 0 |
| CYP706 | 5 | 7 | 11 | 3 | 2 | 4 | 5 | 9 | 0 |
| CYP712 | 4 | 2 | 1 | 2 | 0 | 0 | 9 | 2 | 0 |
| CYP723 | 0 | 0 | 0 | 0 | 0 | 2 | 0 | 0 | 0 |
| CYP736 | 4 | 0 | 8 | 12 | 0 | 0 | 6 | 8 | 0 |
| Others | 0 | 0 | 0 | 0 | 0 | 0 | 0 | 0 | 29 |
| Total | 87 | 154 | 137 | 195 | 143 | 190 | 162 | 166 | 41 |
