# Supplementary figures and images for: Genome-wide identification of the CYP82 gene family in cucumber and functional characterization of CsCYP82D102 in regulating resistance to powdery mildew
Source: PeerJ. 2024 Mar 28;12:e17162. doi: 10.7717/peerj.17162 (PMC10981884; doi:10.7717/peerj.17162)

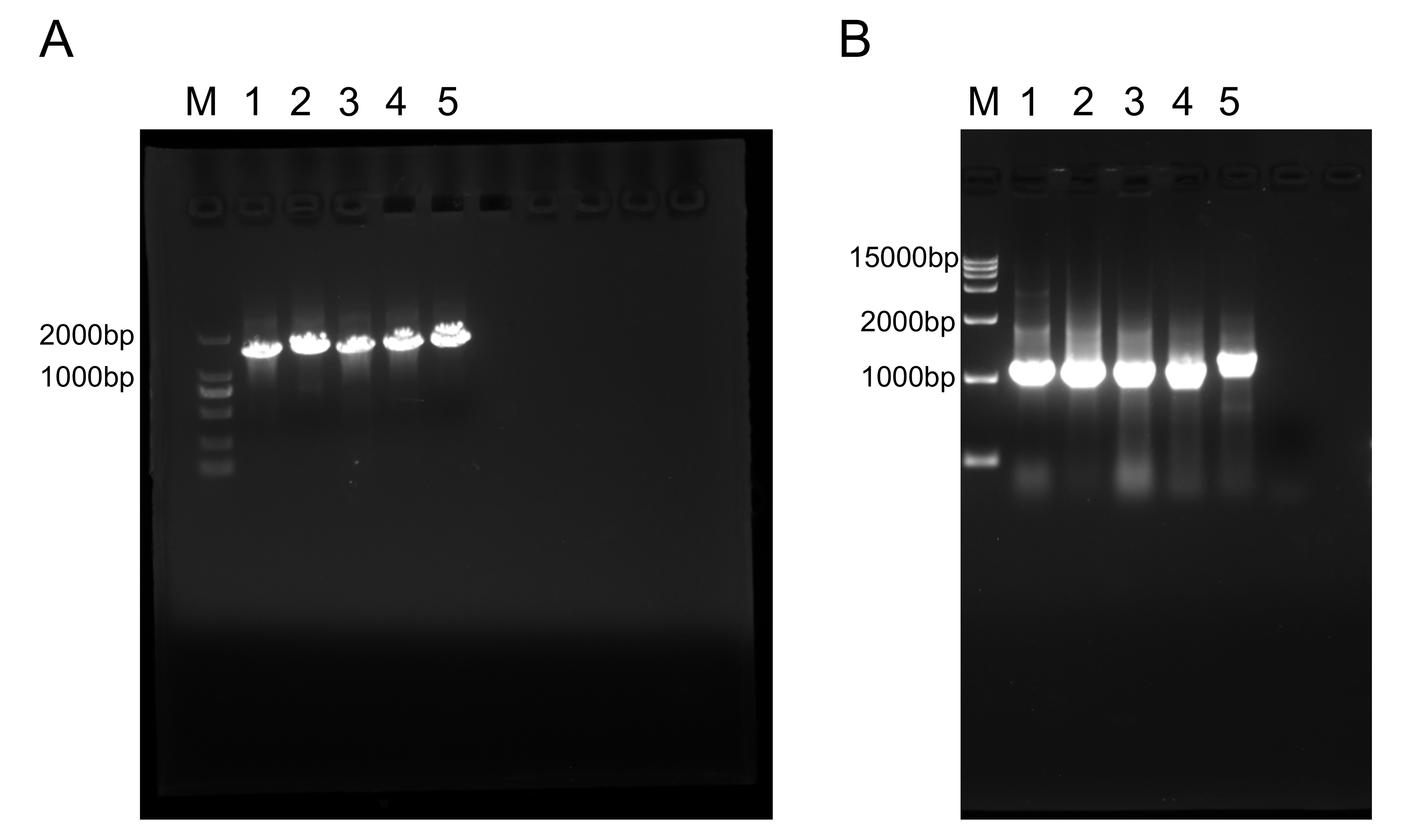

Supplement: Supplemental Information 5 — (A) M: 2000 bp DNA marker; Lane 1: Csa3G853160; Lane 2: Csa3G852590; Lane 3: Csa3G852640; Lane 4: Csa3G852560; Lane 5: Csa3G852600. (B) M: 15000bp DNA marker; Lane 1: Csa3G852400; Lane 2: Csa3G852610; Lane 3: Csa3G852630; Lane 4: Csa3G853140; Lane 5: Csa3G852580. The PCR mixture consisted of 1 μL cDNA template, 1 μL 10 μM Forward primer, 1 μL 10 μM Reverse primer, 12.5 μL 2×ES Taq Master Mix (Dye), and 9.5 μL dd H2O. The PCR verification pictures are shown in Fig. S3. The recommended conditions for PCR were used as follows: 95 °C for 10 min, followed by 40 cycles of 95 °C for 30s, 58 °C for 30s, and 72 °C for 30s. [file peerj-12-17162-s005.png]

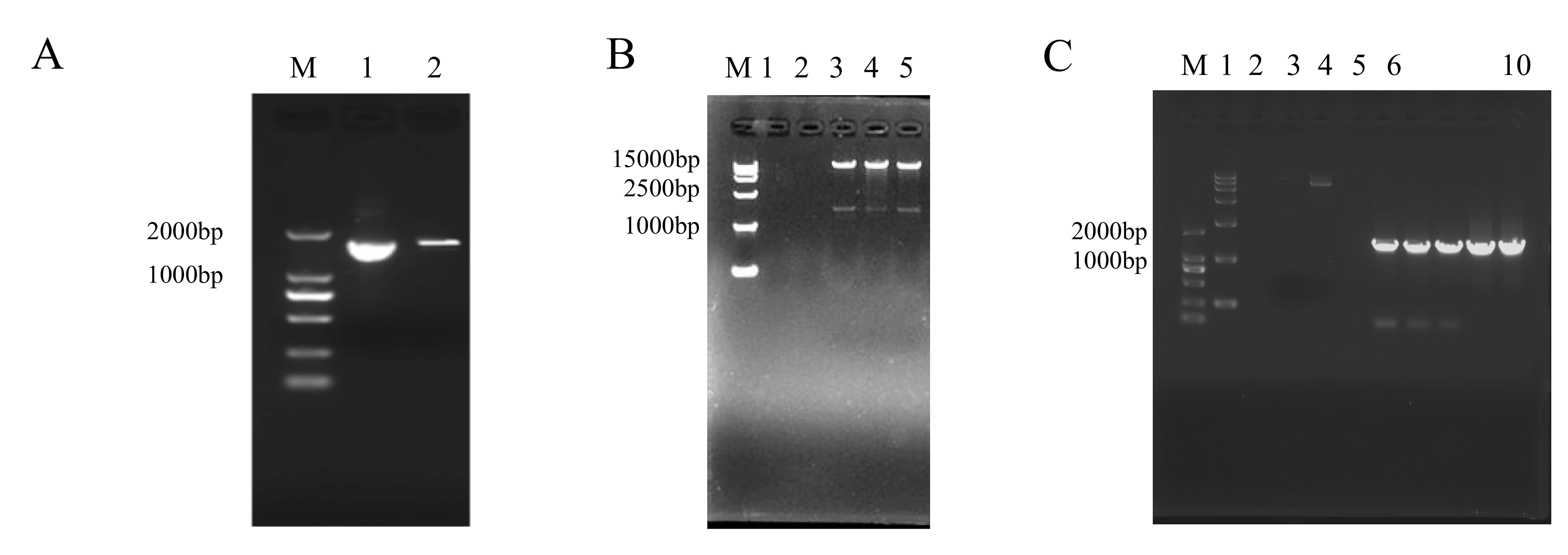

Supplement: Supplemental Information 8 — (A) Gel electrophoresis of PCR products. Lane M displays a 2000 bp DNA marker. (B) Enzymatic double digestion for the identification of CsCYP82D102. Lane M exhibits a 2000 bp DNA marker; Lanes 6-10 depict the PCR amplification of CsCYP82D102. (C) Identification of genetically modified plants. Lane M: 2000 bp DNA marker; Lane 1: 15000 bp DNA marker; Lane 2: water; Lane 3: negative control; Lane 4: LBA4404 plasmids; Lane 6-10: Transgenic plants. [file peerj-12-17162-s008.png]
